# Rate and Characteristics of Metformin Use in Gestational Diabetes

**DOI:** 10.1155/ogi/1243724

**Published:** 2026-08-23

**Authors:** Thanachot Techawijittra, Dittakarn Boriboonhirunsarn

**Affiliations:** ^1^ Department of Obstetrics and Gynecology, Faculty of Medicine Siriraj Hospital, Mahidol University, Bangkok, Thailand, mahidol.ac.th

**Keywords:** dosage, gestational diabetes mellitus, glycemic control, metformin

## Abstract

**Objective:**

To determine the rate and characteristics of metformin use in the treatment of gestational diabetes mellitus (GDM).

**Methods:**

A total of 286 pregnant women diagnosed with GDM at a university‐based tertiary care hospital were included. In addition to nutritional therapy, the decision regarding metformin use was at the physicians’ discretion. Data were retrieved from medical records, including baseline obstetric characteristics, GDM diagnosis, dosage and formulation of metformin, insulin requirement, and pregnancy outcomes. The rate of metformin use was estimated, and factors associated with metformin use were evaluated.

**Results:**

The mean age was 33.8 years, 55.9% were nulliparous, and 43.4% were overweight or obese. Mean gestational age at diagnosis was 18.7 weeks, and 57% were diagnosed before 20 weeks. Metformin was used in 16.1%, with an initial dosage of 500–1000 mg/d in 95.7%. The final dosage was > 1000–2000 mg/d in 43.5%. A dosage increment was required in 50%. Insulin therapy was needed in 13% of those who received metformin. Metformin use significantly increased in those with obesity, high 50‐g glucose challenge test results, and abnormal fasting plasma glucose (FPG) at diagnosis. Logistic regression analysis showed that the only factors that significantly increased the use of metformin therapy were obesity (adjusted OR: 2.88, 95% CI: 1.17–7.07, *p* = 0.021) and abnormal FPG at diagnosis (adjusted OR: 5.06, 95% CI: 2.21–11.57, *p* < 0.001). Maternal and neonatal outcomes were comparable between those with and without metformin use.

**Conclusion:**

Metformin was used in 16.1% of women with GDM. Significant associated factors were obesity and abnormal FPG at diagnosis.

## 1. Introduction

Gestational diabetes mellitus (GDM) is one of the most common pregnancy‐related complications. The global prevalence of GDM averages 14%, with a reported prevalence of 20.8% in the Southeast Asia region [1]. Various adverse pregnancy outcomes have been related to GDM, including preeclampsia, cesarean delivery, infant macrosomia, shoulder dystocia, birth trauma, and neonatal hypoglycemia. It also increases the risk of developing diabetes in both mothers and their children in the future [2, 3]. Evidence shows that adequate glycemic control is key to reducing these complications [4, 5].

Although nutritional therapy is the mainstay for glycemic control in GDM, additional pharmacological therapy is needed when glycemic control is inadequate. Insulin has long been the standard, first‐line pharmacologic therapy for GDM. However, recent evidence suggests that metformin is a safe and effective alternative. Studies demonstrated that metformin improved glycemic control; reduced gestational weight gain (GWG); lowered the risks of GDM‐related complications, such as preeclampsia, cesarean delivery, large‐for‐gestational‐age infants, and macrosomia; and decreased both the need for and dosage of insulin [6–9]. Importantly, current data show no increased risk of congenital anomalies, adverse postnatal growth, or neurodevelopmental impairment [9–11]. In addition, metformin has been shown to be cost‐effective compared to insulin therapy [12]. Many international guidelines recommend the use of metformin as an alternative to insulin or even as a first‐line therapy [2, 13–15]. In Thailand, however, there are currently no recommendations or guidelines regarding the use of metformin in GDM management.

Over the past several years, Siriraj Hospital, the largest university‐based tertiary care hospital in Thailand, has incorporated metformin treatment as an alternative to insulin for glycemic control among women with GDM, yielding favorable results, including continuous reduction in insulin use and improvements in patient satisfaction and compliance.

Nevertheless, there are still limited data on the use of metformin among pregnant women with GDM in various aspects, such as dosage, formulations, associated factors, and pregnancy outcomes. Therefore, the current study primarily aimed to describe the utilization of metformin in women with GDM. The secondary objectives were to identify predictors of its use and to compare pregnancy outcomes between women with and without metformin therapy. This will help improve understanding of current practices and guide the development of appropriate treatment protocols and management strategies for pregnant women with GDM in the future. Moreover, the results could lead to more specific research in this area and to modification of current recommendations at the national level.

## 2. Methods

After approval from the Siriraj Institutional Review Board (SIRB) (COA no. SI1008/2024), a retrospective cohort study was conducted among 286 singleton pregnant women who attended antenatal care at Siriraj Hospital and were diagnosed with GDM in 2024. Exclusion criteria included those with pre‐GDM, fetal anomalies or intrauterine fetal demise, current metformin use, and contraindications for metformin therapy. The sample size was calculated based on an estimated rate of metformin use rate of 15%. At a 95% confidence level and 4.5% allowable error, at least 267 women were required, including a 10% loss.

According to institutional guidelines, GDM screening is offered to all pregnant women at the first antenatal care visit using a 2‐step approach. A 50‐g glucose challenge test (GCT) with a 140 mg/dL cutoff is used as a screening test, and a 100‐g oral GCT (OGTT) is used as a diagnostic test based on Carpenter and Coustan’s criteria. The tests are repeated during 24–28 weeks of gestation if initial test results are normal [16]. As per institutional guidelines, women who are diagnosed before 20 weeks of gestation are classified as early GDM. The term is also used in other studies, and this specific group could have a higher risk of adverse outcomes as previously reported from the same institution and other studies [17–20].

After diagnosis, all women received nutritional counseling from certified diabetes educator nurses, and a follow‐up schedule was set as appropriate. At each follow‐up visit, a fasting plasma glucose (FPG) and/or 2‐h postprandial glucose (2 h PPG) were used to evaluate glycemic control. The glycemic target is set at a FPG of < 95 mg/dL and a 2 h PPG of < 120 mg/dL. If glycemic control is not achieved, pharmacological therapy is advised.

According to institutional recommendation, metformin should be initiated in women with inadequate glycemic control. Medications can be started using either immediate‐ or prolonged‐release formulations at the dosage of 500–1000 mg/d. Dosage and formulation should be evaluated and adjusted every 2‐3 weeks to ensure adequate glycemic control. The maximum recommended dosage is recommended to be 2000 mg/d. The protocol was widely disseminated throughout the Department so that all the caring physicians are informed and to ensure compliance. Although metformin has been incorporated into the management protocol as an alternative to insulin therapy, the decision to use it is based on the physician’s discretion after a thorough discussion with the woman. Insulin therapy is initiated when glycemic control is inadequate after metformin therapy or at the physician’s discretion with endocrinologist consultation.

Data were extracted from medical records, including baseline and obstetric characteristics, GDM screening and diagnosis, use of metformin, need for insulin therapy, and maternal and neonatal outcomes. Details on metformin use were also recorded, such as initial and final dosage and formulation (immediate‐ or prolonged‐release). Metformin failure was defined as the need for insulin therapy in addition to metformin.

Descriptive statistics were used to describe variables as appropriate, including mean, standard deviation, number, and percentage. The rate of metformin use was estimated. Comparisons of various variables were made between those with and without metformin use, using the Student *t*‐test or chi‐square test as appropriate. Logistic regression analysis was used to determine independent associated factors for metformin use, adjusted for potential confounders. Adjusted odds ratios (ORs) and 95% confidence intervals (CIs) were estimated. A *p* value of < 0.05 was considered statistically significant.

## 3. Results

**FIGURE 1:**
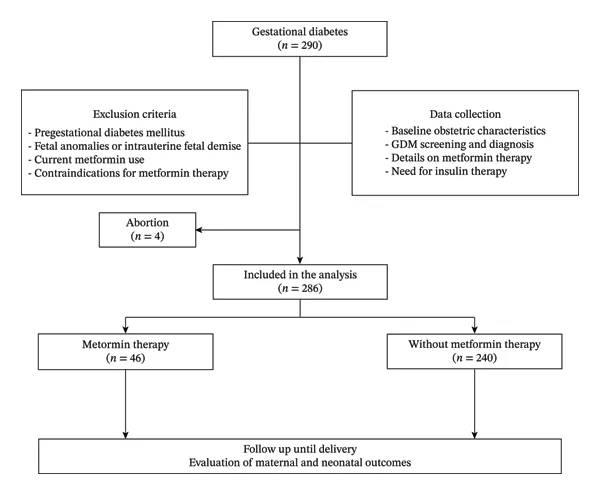
Participant flow diagram.

During 2024, a total of 290 women with GDM were included, but 4 had abortions, leaving 286 women who had complete follow‐up and delivery at the institution in the analysis. The participant flow diagram is shown in Figure 1. Baseline characteristics of the 286 pregnant women diagnosed with GDM included in this study are shown in Table 1. The mean maternal age was 33.8 years, and the mean body mass index (BMI) was 24.9 kg/m^2^. More than half of the participants (55.9%) were nulliparous, and 43.4% were overweight or obese. The most common GDM risk factors were age ≥ 30 years (80.1%), followed by BMI ≥ 25 kg/m^2^ (33.4%) and family history of DM (22.4%).

**TABLE 1:** Baseline characteristics of participants (*N* = 286).

Characteristics	*N* (%)
Mean age ± SD (years)	33.8 ± 5.3
Mean BMI ± SD (kg/m^2^)	24.9 ± 5.1
Nulliparity	160 (55.9)
MI category	
Underweight	16 (5.6)
Normal BMI	146 (51)
Overweight	82 (28.7)
Obese	42 (14.7)
GDM risks	
Age ≥ 30 years	229 (80.1)
BMI ≥ 25 kg/m^2^	124 (43.4)
Familial history of DM	64 (22.4)
Previous GDM	12 (4.2)
Previous macrosomia	1 (0.3)
Previous fetal anomaly	3 (1)
Chronic hypertension	15 (5.2)

Table 2 shows the characteristics of GDM diagnosis among the women. The mean gestational age (GA) at GDM diagnosis was 18.7 ± 8.5 weeks, and the majority (57%) were diagnosed before 20 weeks of gestation (early GDM). The mean 50‐g GCT result was 177.2 mg/dL, 17.1% had a GCT result of ≥ 200 mg/dL, and abnormal FPG (≥ 95 mg/dL) at diagnosis was observed in 12.6%.

**TABLE 2:** Characteristic of GDM diagnosis (*N* = 286).

Characteristics	*N* (%)
Mean GA at diagnosis ± SD (weeks)	18.7 ± 8.5
Mean 50‐g GCT ± SD (mg/dL)	177.2 ± 24.8
High 50‐g GCT (≥ 200 mg/dL)	47 (17.1)
Early GDM (GA < 20 weeks)	163 (57)
Abnormal FPG at diagnosis (≥ 95 mg/dL)	36 (12.6)

Rate and characteristics of metformin use are presented in Table 3. Of the 286 women, 46 (16.1%, 95% CI: 12.0%–20.9%) received metformin treatment. The mean GA at metformin initiation was 25.0 weeks (21.4 ± 6.6 weeks among early GDM and 33.5 weeks among late GDM). Most metformin users (63%, 95% CI: 47.5%–76.8%) had isolated postprandial hyperglycemia (2 h PP ≥ 120 mg/dL), while 4.3% (95% CI: 0.5%–14.8%) had isolated fasting hyperglycemia (FPG ≥ 95 mg/dL), and 32.6% (95% CI: 19.5%–48.0%) had both abnormalities. The initial formulation used was predominantly immediate‐release metformin (71.7%, 95% CI: 56.5%–84.0%), and 43.5% (95% CI: 28.9%–58.9%) of patients received a prolonged‐release formulation as the final treatment. Switching formulations from immediate‐ to prolonged‐release was observed in 15.2% (95% CI: 6.3%–28.9%) of cases. Nearly all patients (95.7%, 95% CI: 85.2%–99.5%) started metformin with a dose of 500–1000 mg/day, and 43.5% (95% CI: 28.9%–58.9%) required a dose of > 1000–2000 mg/day as the final treatment. An increase in metformin dosage was observed in 50% of cases. Of all GDM women, insulin therapy was required in 10 women (3.5%, 95% CI: 10.9%–36.4%). Among these, 4 women received insulin without prior metformin therapy, while 6 women received metformin, corresponding to a 13% (95% CI: 4.9%–26.3%) metformin failure rate.

**TABLE 3:** Rate and characteristics of metformin use (*N* = 46).

Characteristics	*N* (%)
Rate of metformin use	46 (16.1)
Mean GA at metformin initiation ± SD (weeks)	25.0 ± 8.0
Early GDM	21.4 ± 6.6
Late GDM	33.5 ± 2.5
FPG and 2 h PPG at metformin initiation	
High FPG only (≥ 95 mg/dL)	2 (4.3)
High 2 h PPG only (≥ 120 mg/dL)	29 (63)
High both FPG and 2 h PPG	15 (32.6)
Initial metformin formulation use	
Metformin immediate‐release	33 (71.7)
Metformin prolonged‐release	13 (28.3)
Final metformin formulation use	
Metformin immediate‐release	26 (56.5)
Metformin prolonged‐release	20 (43.5)
Metformin formulation change^∗^	
Remained with immediate‐release	26 (56.5)
Remained with prolonged‐release	13 (28.3)
From immediate‐ to prolonged‐release	7 (15.2)
Additional insulin use	6 (13)
Initial metformin dose	
500–1000 mg/d	44 (95.7)
> 1000–1500 mg/d	2 (4.3)
Final metformin dose	
500–1000 mg	26 (56.5)
> 1000–2000 mg	20 (43.5)
Metformin dose increase	23 (50)

^∗^No women switched treatment from immediate‐ to prolonged‐release formulation or used the combination of the 2 formulations.

Comparisons were made between women with and without metformin therapy, and the results are shown in Table 4. Women receiving metformin therapy had a significantly higher BMI (27.6 vs. 24.4 kg/m^2^, *p* = 0.002) and were more commonly to be obese (30.4% vs. 11.7%, *p* = 0.001). They also had significantly higher mean 50‐g GCT values (*p* = 0.003) and a higher proportion of abnormal FPG results (*p* < 0.001).

**TABLE 4:** Comparisons of characteristics between GDM women with and without metformin use.

Characteristics	Metformin use		*p* value
	No (*N* = 240)	Yes (*N* = 46)	
Mean age ± SD (years)	34 ± 5.2	32.4 ± 5.7	0.051
Mean BMI ± SD (kg/m^2^)	24.4 ± 4.7	27.6 ± 6.3	0.002
Parity			0.145
Nulliparity	139 (57.9%)	21 (45.7%)	
Multiparity	101 (42.1%)	25 (54.3%)	
BMI category			0.001
Normal/underweight	145 (60.4%)	17 (36.9%)	
Overweight	67 (27.9%)	15 (32.6%)	
Obese	28 (11.7%)	14 (30.4%)	
Mean GA at diagnosis ± SD (weeks)	19.2 ± 8.4	16.5 ± 8.6	0.051
Mean 50‐g GCT ± SD (mg/dL)	175.3 ± 24.2	187.2 ± 25.8	0.003
Timing of diagnosis			0.06
Early GDM (GA < 20 weeks)	131 (54.6%)	32 (69.6%)	
Late GDM	109 (45.4%)	14 (30.4%)	
50‐g GCT at diagnosis			0.079
< 200 mg/dL	203 (84.5%)	34 (73.9%)	
≥ 200 mg/dL	37 (15.4%)	12 (26.1%)	
FPG at diagnosis			< 0.001
Normal FPG	221 (92.1)	29 (63.0)	
Abnormal FPG	19 (7.9)	17 (37.0)	

Logistic regression analysis was performed to determine independent factors that predict the use of metformin, and the results are demonstrated in Table 5. After adjustment for confounders, independent factors that increased the risk of metformin therapy were obesity (adjusted OR: 2.88, 95% CI: 1.17–7.07, *p* = 0.021) and abnormal FPG at diagnosis (adjusted OR: 5.06, 95% CI: 2.21–11.57, *p* < 0.001).

**TABLE 5:** Logistic regression analysis to determine independent factors associated with metformin use.

Variables	Adjusted OR	95% CI	*p* value
Age > 30 years	0.56	0.27–1.18	0.128
BMI category			
Normal/underweight	1.0		
Overweight	1.45	0.65–3.26	0.366
Obese	2.88	1.17–7.07	0.021
Early GDM	0.55	0.26–1.17	0.121
High 50‐g GCT ≥ 200 mg/dL	0.91	0.37–2.27	0.845
Abnormal FPG at diagnosis	5.06	2.21–11.57	< 0.001

Table 6 shows the comparison of obstetric and neonatal outcomes between women with and without metformin use. Those with metformin use had lower GWG without statistical significance, but they were significantly more likely to exhibit excessive GWG (35.7% vs. 19.5%, *p* = 0.014). Other maternal and neonatal outcomes were comparable, including GA at delivery, route of delivery, preeclampsia, birth weight, LGA, macrosomia, Apgar scores, neonatal hypoglycemia, and NICU admission.

**TABLE 6:** Comparisons of pregnancy outcomes between GDM women with and without metformin use.

Characteristics	Metformin use		*p* value
	No (*N* = 240)	Yes (*N* = 46)	
*Maternal outcomes*			
Mean GA at delivery ± SD (weeks)	37.9 ± 1.6	37.7 ± 1.3	0.488
Mean gestational weight gain ± SD (kg)	10.2 ± 5.1	8.7 ± 6.5	0.09
Gestational weight gain category			0.014
Inadequate weight gain	94 (43.7%)	20 (47.6%)	
Normal weight gain	79 (36.7%)	7 (16.7%)	
Excessive weight gain	42 (19.5%)	15 (35.7%)	
Route of delivery			0.715
Vaginal delivery	72 (33.5%)	12 (28.6%)	
Primary cesarean delivery	93 (43.3%)	21 (50%)	
Repeat cesarean delivery	50 (23.3%)	9 (21.4%)	
Preeclampsia	11 (4.6%)	4 (8.7%)	0.253

*Neonatal outcomes*			
Mean birth weight ± SD (g)	2987.5 ± 495.8	3032.9 ± 479.2	0.586
Birth weight for GA			0.575
SGA	33 (15.3%)	6 (14.3%)	
AGA	141 (65.6%)	25 (59.5%)	
LGA	41 (19.1%)	11 (26.2%)	
Macrosomia	4 (1.9%)	1 (2.4%)	0.593
Apgar < 7 at 1 min	12 (5.6%)	5 (11.9%)	0.167
Apgar < 7 at 5 min	2 (0.9%)	0 (0%)	1.0
Phototherapy	26 (12.1%)	9 (21.4%)	0.137
Neonatal hypoglycemia	6 (2.8%)	3 (7.1%)	0.168
NICU admission	15 (7%)	5 (11.9%)	0.34

## 4. Discussion

The results showed that the rate of metformin use in this population was 16.1%. This could reflect the proportion of GDM women whose glycemic control was inadequate with nutritional therapy. When compared with the previous rate of insulin use prior to the integration of metformin into management, the rate of metformin use was relatively higher than the insulin use rate of 3%–9% reported in previous studies from the same institution [16, 19, 21]. This could be due to the fact that metformin is more easily accepted by the women than insulin. As a result, physicians were more likely to make earlier decisions to initiate pharmacological therapy.

There is currently no standard recommendation of metformin dosage and formulation in GDM women. The results demonstrated that the initial prescribed dosage formulation used was 500–1000 mg/d in 95.7% of cases, with 71.7% using the immediate‐release formulation. An increase in metformin dosage to achieve the glycemic target was observed in 50% of cases, and a final dosage of > 1000–2000 mg/d is needed in 43.5%. The maximum use of metformin dosage of 2500 mg/d has been reported as safe in previous major clinical trials without serious adverse events [6, 7]. Switching formulations from immediate‐ to prolonged‐release was observed in 15.2% of cases. The change in formulation, accompanied by an increased dosage, could be due to the fewer side effects associated with prolonged‐release formulations, which may also help improve treatment compliance [22, 23].

The findings demonstrated that GDM women who required metformin therapy were significantly more likely to be obese, have high GCT results, and abnormal FPG at diagnosis. However, multivariate logistic regression analysis showed that only obesity and abnormal FPG significantly increased the chance of metformin therapy by approximately 3 and 5 times, respectively. These risk factors were similar to those of insulin use previously reported in various studies [24–29]. As demonstrated in previous studies, high prepregnancy BMI has been associated with increased insulin resistance [30, 31]. As a result, this group of women was less likely to achieve adequate glycemic control with only nutritional therapy. In addition, high GCT and abnormal FPG at the time of diagnosis reflected a higher degree of glucose intolerance among these women, which resulted in the additional need for pharmacological therapy. These predictive factors might be useful as early markers for identifying GDM women who are at higher risk of failing lifestyle and nutritional management.

Failure of metformin for glycemic control in this study was 13%. Variations in the metformin failure rate have been reported in many previous studies, ranging from 15% to 36% [32–34]. This could be due to differences in study populations, GDM diagnoses, and treatment guidelines. The low failure rate observed in this study could also be attributed to the intensive counseling provided to the women at each visit. Risk factors for metformin failure that have been consistently reported include high FPG [32–34] and high prepregnancy BMI [34], which reflect the risk of greater insulin resistance. As per institutional practice, awareness is raised among physicians, and these at‐risk women were scheduled for close follow‐up and monitoring, such as every 1‐2 weeks, to evaluate glycemic control. This could help physicians to make timely decisions to determine metformin failure and initiate additional insulin therapy to achieve adequate glycemic control. It should be noted that there were 4 women who received insulin therapy without prior metformin, and some of them could possibly have benefited from avoiding insulin treatment if metformin had been initially prescribed. As there is limited evidence on metformin failure and its predictors, these results provide additional information from a real‐world clinical practice in a tertiary care setting which could be useful for future investigations on this important issue.

The results also showed that the majority of pregnancy outcomes were not significantly different between GDM with and without metformin therapy. While GWG was slightly lower among those with metformin therapy, the rate of excessive GWG was significantly higher. This could be because, although metformin has been reported to help in GWG control [6, 7], it was more commonly used among overweight or obese women who were more likely to exhibit excessive GWG. In addition, those with excessive GWG were also less likely to achieve a glycemic target. Although neonatal outcomes were comparable between the 2 groups, safety issues need to be confirmed by future studies.

The strengths of this study might include the uniform practice of institutional guidelines in GDM screening and diagnosis, and all GDM women received similar, individually customized counseling by certified diabetes educator nurses at every visit. The study reported real‐world clinical data from a tertiary setting with practical insights into metformin dosing and formulation. This is highly relevant for regions where standardized GDM pharmacotherapy guidelines are still evolving.

However, some limitations should be noted. The nature of retrospective data collection could result in inaccurate or incomplete information. Nevertheless, all medical records were thoroughly reviewed, and questionable information was clarified and confirmed from other related sources. The decision to initiate metformin therapy and dosage adjustment was not mandatory and was based mainly on physicians’ decisions, given that there are still no standard national or international guidelines on this issue. This could be subject to selection bias, but this should be minimal, as the protocol for metformin therapy has been widely distributed throughout the department, so all the physicians were informed and more likely to comply. It can also be noted that only 4 women were offered insulin therapy without metformin. The study also has limited power in evaluating differences between groups since there were only 46 women who received metformin therapy. This issue should be further evaluated in future, larger studies. A single‐center study could also restrict the generalizability to other populations.

As the effectiveness and safety of metformin use for glycemic control in GDM have been consistently reported, its use should be considered by obstetricians, especially in Thailand, where metformin use in GDM is limited. A standardized guideline on metformin use should be developed at both national and international levels to provide support and increase confidence among healthcare personnel and pregnant women. Further studies are warranted in order to explore in more detail the most appropriate dosage and formulations, treatment timing, follow‐up schedule, and risks of treatment failure in various populations and settings.

## 5. Conclusion

In conclusion, the rate of metformin use for glycemic control among women with GDM was 16.1%, and additional insulin therapy was needed in 13%. Prepregnancy obesity, high 50‐g GCT results, and abnormal FPG at the time of diagnosis were significantly associated with metformin use. Pregnancy outcomes were comparable between those with and without metformin use.

## Funding

The authors have nothing to report.

## Disclosure

The research has been accepted to present as an e‐poster at the 15^th^ International Congress of Diabetes and Metabolism (ICDM 2025) during 25–27 September, 2025, at the Grand Walkerhill, Seoul, Republic of Korea [35].

## Conflicts of Interest

The authors declare no conflicts of interest.

## Data Availability

The data that support the findings of this study are available from the corresponding author upon reasonable request.
